# Maternal Folate Receptor Alpha Autoantibodies and Increased Fetal Nuchal Translucency as Potential Early Markers of Autism Spectrum Disorder

**DOI:** 10.1002/brb3.71088

**Published:** 2025-11-21

**Authors:** Claudio Giorlandino, Katia Margiotti, Marco Fabiani, Alvaro Mesoraca, Laura D'Emidio, Raffaella Raffio, Claudio Coco, Maria Luisa Mastrandrea, Chiara Pasquale, Marina Cupellaro, Francesca Giorlandino, Francesco Pignataro, Vincenzo Milite

**Affiliations:** ^1^ Human Genetics Lab, Altamedica Main Centre Rome Italy; ^2^ Department of Prenatal Diagnosis Altamedica, Fetal‐Maternal Medical Centre Rome Italy

## Abstract

**Purpose:**

To investigate the association between increased fetal nuchal translucency (NT) and maternal folate receptor alpha autoantibodies (FRAA) positivity, and to evaluate the subsequent risk of non‐syndromic autism spectrum disorder (ASD) in offspring.

**Methods:**

A total of 3600 first‐trimester ultrasounds were screened at a fetal medicine center. Among these, 27 fetuses with markedly increased NT (≥ 3.5 mm) underwent invasive prenatal diagnosis, including karyotyping, CGH array, and postnatally whole‐exome sequencing (WES) when standard tests were negative. Maternal serum samples were tested for FRAA using ELISA. Eleven pregnancies with negative genetic testing were followed longitudinally, and neurodevelopmental outcomes in children were assessed up to 36 months using ADOS‐2 and DSM‐5 criteria.

**Findings:**

Among the 11 fetuses with negative genetic outcomes, 4 mothers tested positive for FRAA. All four FRAA‐positive offspring were later diagnosed with ASD, while only one of the seven FRAA‐negative offspring received an ASD diagnosis. FRAA‐positive cases exhibited markedly increased NT (≥ 3.5 mm) but no pathogenic genetic variants, suggesting an immune‐mediated etiology. FRAA levels persisted in maternal and neonatal serum, implying ongoing exposure during gestation.

**Conclusion:**

FRAA positivity in pregnancies with isolated markedly increased NT may serve as an early biomarker of increased ASD risk in offspring. These findings support the hypothesis of an immune‐metabolic mechanism contributing to ASD and suggest potential preventive interventions such as folinic acid supplementation.

## Graphical abstract

Maternal folate receptor alpha autoantibodies (FRAA) were associated with isolated increased fetal nuchal translucency and subsequent autism spectrum disorder (ASD) in offspring. FRAA positivity may represent an early biomarker of neurodevelopmental risk, suggesting potential preventive strategies such as folinic acid supplementation.

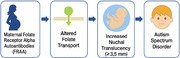

## 1 Introduction

An increased nuchal translucency (NT) measurement during the first trimester is a well‐established ultrasound marker linked to a wide spectrum of fetal abnormalities. Traditionally, enlarged NT has been strongly associated with chromosomal aneuploidies, congenital heart defects, and genetic syndromes (Socolov et al. 2017, Snijders et al. 1998, Baer et al. 2014, Souka et al. 1998). More recently, emerging evidence has suggested a potential relationship between enlarged NT and neurodevelopmental conditions, including non‐syndromic autism spectrum disorder (ASD) (Hellmuth et al. 2017, Lithner et al. 2016, Ayras et al. 2015).

The prevalence of ASD has risen markedly, with recent estimates indicating that approximately 1 in 36 children are affected (Bobrowski‐Khoury et al. 2021). Growing evidence implies not only genetic but also immune‐mediated mechanisms in the pathogenesis of ASD, particularly during fetal development (Zhuang et al. 2020, Frye et al. 2013, Frye et al. 2022).

In particular, autoantibodies against the folate receptor alpha (FRAA) have been shown to interfere with folate transport across the placental and blood‐brain barriers, impairing folate delivery to the fetal central nervous system and increasing the risk of ASD and cognitive impairments in offspring (Frye et al. 2022, Frye et al. 2018, Ramaekers and Quadros 2022). Interestingly, preclinical and clinical studies suggest that folinic acid supplementation in FRAA‐positive pregnant women can improve neuropsychiatric outcomes in their children, reducing the incidence of ASD and learning disabilities (Frye et al. 2018, Rossignol and Frye 2012, Renard et al. 2020, Giorlandino et al. 2025).

In this study, we investigated the presence of folate receptor alpha autoantibodies (FRAA) in pregnant women carrying fetuses with markedly increased nuchal translucency (NT > 3.5 mm), a known risk factor for neurodevelopmental disorders. Our aim was to evaluate whether FRAA positivity may represent an early prenatal biomarker of increased ASD risk and to explore the potential preventive effect of folinic acid supplementation in such high‐risk pregnancies.

## 2 Material and Methods

Between January 2021 and December 2023, approximately 3600 first‐trimester ultrasound examinations were carried out at the Altamedica Fetal Medicine Center. Written informed consent was obtained from all mothers for participation in the study, including collection of biological material, genetic testing, and follow‐up evaluation. Of these, 108 fetuses showed an increased nuchal translucency (NT > 2.5 mm), and 27 cases presented with a markedly increased NT (≥ 3.5 mm). Pregnancies with elevated NT were referred for invasive prenatal testing, performed by amniocentesis or chorionic villus sampling (CVS), depending on gestational age and maternal preference. Standard investigations included conventional karyotyping and, when required, comparative genomic hybridization (CGH) array.

Among the 27 cases with markedly increased NT (≥ 3.5 mm), all families were invited to participate in a dedicated research protocol for the evaluation of folate receptor alpha autoantibodies (FRAA). The protocol included invasive genetic testing, such as karyotyping, CGH array, and postnatal WES when indicated. In 11 of these 27 cases, maternal blood samples were obtained at delivery, and follow‐up samples were collected from the offspring at 6 months of age.

Serum samples were screened for FRAA using a commercial enzyme‐linked immunosorbent assay (ELISA), as previously described (Giorlandino et al. 2025). FRAA positivity was defined as concentrations ≥ 30 ng/ml in both maternal and neonatal samples.

WES was performed using the VAHTS Target Capture Core Exome Panel (Vazyme, China), following the manufacturer's protocol. This panel covers 19,441 genes included in RefSeq, CCDS, and ClinVar. Sequencing achieved an average coverage of 80×, with > 98% of target bases covered at ≥ 30 × depth. Detected variants were annotated and filtered using functional prediction algorithms (PolyPhen2, SIFT, REVEL), disease databases (ClinVar, HGMD, OMIM, GWAS), and population allele frequency repositories (dbSNPs, ALFRED, gnomAD, ExAC, 1000 Genomes). Variant classification was performed using commercial software (enGenome—eVAI, v3.5, CE‐IVD). Variants meeting ACMG/AMP criteria for pathogenicity, likely pathogenicity, and high‐confidence variants of uncertain significance (VUS) related to relevant Human Phenotype Ontology (HPO) terms such as HP:0001249 (Intellectual disability), HP:0000717 (Autism), HP:0001263 (Global developmental delay), HP:0000750 (Delayed speech and language development), HP:0011098 (Speech apraxia), and HP:0002546 (Incomprehensible speech), were invetigated.

CGH array was performed using the 44K platform (Agilent Technologies, Santa Clara, CA) to exclude established genetic etiologies associated with neurodevelopmental disorders.

Neurodevelopmental evaluation for autism spectrum disorder (ASD) was carried out with the Autism Diagnostic Observation Schedule, Second Edition (ADOS‐2), a widely accepted semi‐structured assessment tool for diagnosing autism (Lord et al. 2020). Examinations were conducted by trained clinicians blinded to maternal FRAA results, and all diagnoses were confirmed according to DSM‐5 criteria (Mazefsky et al. 2013).

## 3 Results

Between January 2021 and December 2023, approximately 3,600 first‐trimester ultrasound examinations, performed between 11 + 0 and 13 + 6 weeks of gestation, were conducted at the Altamedica Fetal Medicine Center. Among these, 108 fetuses (3.0%) presented with increased nuchal translucency (NT > 2.5 mm), and 27 cases (0.75%) had markedly increased NT (≥ 3.5 mm). All fetuses with markedly NT ≥ 3.5 mm underwent chorionic villus sampling (CVS) or amniocentesis followed by conventional karyotyping and CGH array analysis. In our cohort, none of the mothers had autoimmune disorders, and all were under standard folate supplementation during pregnancy.

**FIGURE 1:**
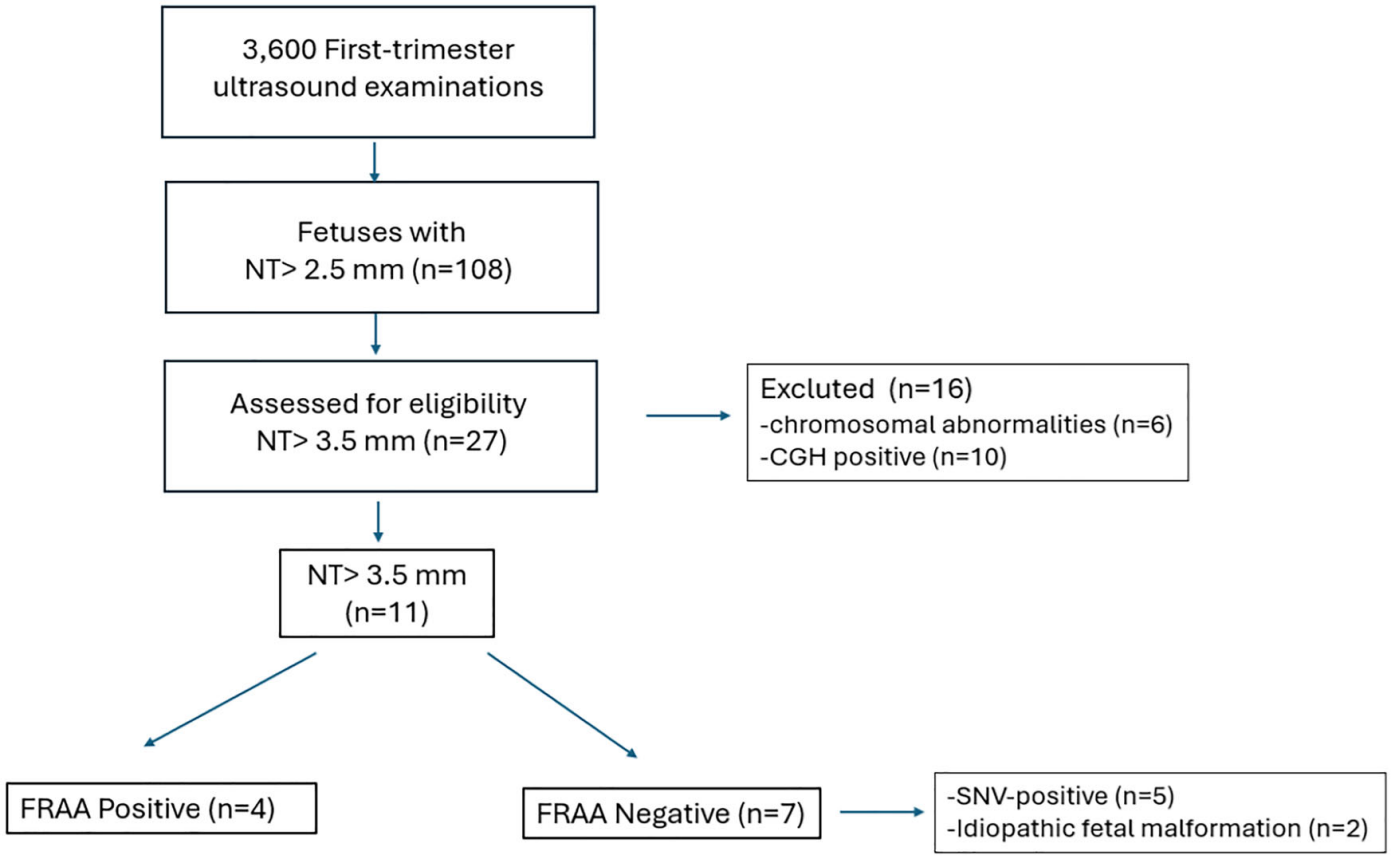
CONSORT flow diagram of the study population.

Of these 27 cases, 16 were found to have chromosomal abnormalities or pathogenic copy number variants (CNVs), and pregnancies were electively terminated (Figure 1). Postnatally, t he remaining 11 cases underwent trio whole‐exome sequencing, and mothers were analyzed using a CE‐IVD–validated ELISA for both blocking and binding folate receptor alpha autoantibodies (FRAA).

In our cohort, we did not observe substantial differences in baseline characteristics between the included (*n* = 11) and excluded pregnancies (*n* = 16); all women belonged to a homogeneous obstetric population, with comparable maternal age (28–39 years), parity (0–2), comorbidities (none beyond minor gestational conditions), and folate supplementation (all under standard treatment during pregnancy). The final 11 cases consisted exclusively of fetuses with markedly increased NT (≥ 3.5 mm) and normal detailed anatomic and echocardiographic assessments, that is, isolated NT.

FRAA positivity was defined as serum titers above the 97.5th percentile (≥ 30  ng/mL) (Giorlandino et al. 2025). FRAA positivity was detected in four out of 11 mothers. Among the four cases in which the mothers were FRAA‐positive, genetic analysis of the newborn revealed no pathogenic or likely pathogenic single‐nucleotide variants (SNVs). Furthermore, all four children exhibited normal postnatal morphology without major congenital anomalies. Conversely, among the 7 FRAA‐negative cases, pathogenic SNVs were detected in five newborn, providing a clear genetic diagnosis. The remaining two cases in this group remained undiagnosed and presented mild malformations of unknown genetic origin. These 11 infants remained under our pediatric care and thus constituted a convenience sample, rather than an incidence‐based cohort. These findings highlight a distinct separation: FRAA‐positive cases lacked identifiable genetic or structural abnormalities, whereas the majority of FRAA‐negative cases had alternative genetic explanations for their outcomes. Table 1 summarizes the clinical and genetic characteristics of the four cases with maternal FRAA positivity and the seven with maternal FRAA negativity. All four fetuses exhibited significantly increased nuchal translucency, with a mean NT of 4.1 mm (range: 3.6–4.6 mm), yet standard karyotype and chromosomal microarray analyses were normal in each case.

**TABLE 1:** Summary of the clinical and genetic findings in the case series (*N* = 11).

Parameter	FRAA‐positive	FRAA‐negative
**Number of cases**	4	7
**Mean nuchal translucency (NT)**	4.1 mm (range 3.6–4.6 mm)	3.9 mm (range 3.2–4.1 mm)
**Karyotype and array‐CGH**	Normal in all cases	Normal in all cases
**Trio exome sequencing**	No pathogenic variants detected	5 patogenic variants in: *POMT1*, *FGFR3*, *CEP290*, *DHCR7*, *RAF1*.
**Postnatal ASD diagnosis**	ADOS‐2 (11–16) and DSM‐5 criteria positive	1 child had an ADOS‐2 0 =11 and DSM‐5 criteria positive

Furthermore, trio‐based whole‐exome sequencing revealed no pathogenic or likely pathogenic variants in the FRAA‐positive group, effectively excluding known monogenic causes of neurodevelopmental disorders. In contrast, five pathogenic variants were identified in the FRAA‐negative group, involving *POMT1*, *FGFR3*, *CEP290*, *DHCR7*, and *RAF1*. Notably, FRAA levels were found to be elevated in both maternal serum and in the neonatal serum at 6 months of age in the FRAA‐positive group, suggesting persistent autoantibody exposure during gestation (Table 1).

ASD assessment was feasible only for the 11 children who remained under follow‐up at our center, as systematic neurodevelopmental evaluation (including ADOS‐2 testing) could not be performed for the other cases due to elective termination of pregnancy. Among them, all four children born to FRAA‐positive mothers were diagnosed with ASD between 24 and 36 months of age, based on standardized ADOS‐2 scores (range 11–16) and DSM‐5 criteria. Among the seven FRAA‐negative cases, and taking into account the genetic conditions identified in this subgroup, only one child showed a clearly defined autistic profile with an ADOS‐2 score = 11 and meeting DSM‐5 diagnostic criteria for ASD, whereas the remaining six did not meet the criteria during neurodevelopmental follow‐up (Table 1) (Lord et al. 2020, Mazefsky et al. 2013).

Thus, in our cohort, the prevalence of ASD was markedly higher among children born to FRAA‐positive mothers (100%) compared to those born to FRAA‐negative mothers (14.3%). For all 11 infants, standardized neurodevelopmental assessment was performed using ADOS‐2 scores and DSM‐5 criteria, ensuring uniform and systematic screening for ASD within the cohort.

## 4 Discussion

Our findings suggest that markedly increased NT in the absence of chromosomal, structural, or single‐gene anomalies may, in certain cases, reflect an underlying immuno‐metabolic mechanism rather than a purely anatomical or genetic etiology. In particular, the detection of maternal FRAA, predominantly of the IgG class, in pregnancies with euploid fetuses presenting isolated increased NT, raises the possibility of a pathophysiological link between altered folate bioavailability and transient fetal edema.

FRAA has been shown to cross the placenta and interfere with fetal folate transport by targeting folate receptor alpha (*FOLR1*), which mediates cellular uptake of 5‐methyltetrahydrofolate, the biologically active form of folate (Bobrowski‐Khoury et al. 2021). The importance of this receptor in embryonic development is underscored by the embryonic lethality observed in *Folr1* knockout mice (Sangha et al. 2022), as well as by its predominant expression in the neural epithelium and its apical localization during neural tube closure (Piedrahita et al. 1999, Barber et al. 1999, Saitsu et al. 2003). Recent studies have extended the functional role of *FOLR1* beyond folate transport, demonstrating its involvement in epithelial morphogenesis through the regulation of apical constriction, a process crucial for neural tube closure and tissue remodeling. Folic acid has been shown to promote apical constriction via a *FOLR1‐* and MLCK‐dependent mechanism, independently of Rho‐kinase signaling (Martin et al. 2019). Moreover, in vivo and in vitro models have demonstrated that *FOLR1* regulates cytoskeletal organization and cellular polarity during neurulation, contributing directly to morphogenetic processes critical for early embryonic development (Martin et al. 2019).

These findings offer a plausible biological explanation for the transient cervical edema underlying increased NT in our cohort. Disruption of *FOLR1* signaling by maternal FRAA may impair apical constriction in epithelial tissues and interfere with lymphatic development and interstitial fluid drainage, both of which are essential for the resolution of physiological fetal edema during the first trimester. Importantly, all children in our study cohort who presented with isolated increased NT and FRAA positivity were later diagnosed with ASD by the age of three, suggesting a potential association between FRAA exposure and neurodevelopmental risk; however, these observations should be considered preliminary and hypothesis‐generating rather than definitive evidence of early biomarkers or efficacy of preventive strategies.

Taken together, our results support the hypothesis that maternal FRAA can interfere with folate‐dependent pathways crucial for fetal development through both metabolic and morphogenetic mechanisms. This finding supports exploring preventive strategies—for example, targeted screening and high‐dose folinic acid supplementation in FRAA‐positive pregnancies, especially with isolated increased NT—as hypotheses for future controlled trials. Any mention of prevention will be restricted to evidence derived from randomized studies. Limitations include the small sample size, retrospective design, and absence of maternal FRAA titers in early pregnancy. From a statistical standpoint, the small sample yields wide confidence intervals and limited generalizability; accordingly, these preliminary, hypothesis‐generating findings should be interpreted as pilot data. Nevertheless, the uniformity of the phenotype (isolated NT, normal genetics, FRAA positivity, ASD) across all four cases strengthens the plausibility of a shared pathophysiological pathway. Prospective multicenter studies are warranted to (i) determine the prevalence of FRAA among pregnancies with isolated NT, (ii) chart longitudinal antibody kinetics, and (iii) evaluate targeted folate‐based interventions. Among the most intriguing hypotheses is that if FRAA testing were systematically performed in all cases of increased NT, it might be possible to modulate the effects on neurodevelopment through folinic acid supplementation in many additional cases, considering the high prevalence of non‐syndromic autism (Giorlandino et al. 2025).

## 5 Conclusions

To our knowledge, this brief series is the first to suggest an association between markedly increased nuchal translucency with negative comprehensive genetic testing and mother–child FRAA positivity with later non‐syndromic ASD. NT may therefore be viewed as an early candidate imaging signal of maternal‐fetal immune dysregulation. Recognizing this pathway could inform timely screening strategies and the design of preventive interventions—such as high‐dose folinic acid supplementation—to be tested in prospective, randomized trials rather than recommended at this stage.

A total of 3600 first‐trimester ultrasound examinations were performed. Among these, 108 fetuses with increased NT > 2.5 mm were identified. Of these, 27 fetuses with markedly increased NT > 3.5 mm were assessed for eligibility. Sixteen cases were excluded due to the presence of chromosomal abnormalities (*n* = 6) or positive CGH findings (*n* = 10). The remaining 11 cases were subdivided as follows: 4 FRAA‐positive, 7 FRAA‐negative (including 5 SNV‐positive and 2 mild idiopathic fetal malformations).

### 5.1 What's Already Known About This Topic?

Recent literature has linked isolated increased NT with neurodevelopmental disorders, including non‐syndromic ASD.

Folate receptor alpha autoantibodies (FRAA) have been implicated in the disruption of folate transport across the placenta and blood‐brain barrier, contributing to ASD pathogenesis.

### 5.2 What Does This Study Add?

This is the first study to report an association between isolated increased NT, maternal FRAA positivity, and later ASD diagnosis in the offspring.

The study proposes a novel immune‐metabolic mechanism for increased NT, potentially linked to disrupted folate signaling and morphogenesis.

## Author Contributions

Conceptualization, C.G., A.M.; methodology K.M.; validation A.M., M.C.; patient clinical management L.D.E., R.R., C.C., and C.P.; C.G. F.P., and V.M. final draft preparation and critical review. All authors have read and agreed to the published version of the manuscript.

## Funding

The authors have nothing to report.

## Conflicts of Interest

The authors declare no conflicts of interest.

## Ethics Statement

The study was approved by the Ethics Committee of Artemisia S.p.A., Rome, Italy, on [January 10, 2021], with approval number [# 2021/45].

## Consent

Informed consent was obtained from subjects involved in the study.

## Data Availability

Data is available upon reasonable request.
